# Clinical and epidemiological comparison between perimesencephalic and non-perimesencephalic, non-aneurysmal subarachnoid hemorrhage: a single-center cohort study from a referral center in a developing country

**DOI:** 10.1055/s-0046-1827159

**Published:** 2026-08-13

**Authors:** João Pedro Einsfeld Britz, Rafael Harter Tomaszeski, Carla Bittencourt Rynkowski, Diego Zambonin, Ericson Sfreddo, Paulo Valdeci Worm

**Affiliations:** 1 Grupo Hospitalar Conceição, Hospital Cristo Redentor, Departamento de Neurocirurgia, Porto Alegre RS, Brazil.; 2 Grupo Hospitalar Conceição, Hospital Cristo Redentor, Unidade de Cuidado Intensivo, Porto Alegre RS, Brazil.

**Keywords:** Subarachnoid Hemorrhage, Intracranial Hemorrhages, Cerebrovascular Disorders, Stroke

## Abstract

**Background:**

Non-aneurysmal subarachnoid hemorrhage (SAH) represents a heterogeneous entity with implications for management and prognosis. Perimesencephalic SAH (PSAH) is associated with a more benign clinical course, lower rates of complications, and favorable outcomes, whereas non-perimesencephalic SAH (NPSAH) tends to present with higher rates of vasospasm, hydrocephalus, and worse neurological outcomes. Recognizing these distinct bleeding patterns is, therefore, essential for clinical management.

**Objective:**

The present study aims to analyze a series of patients with nonaneurysmal SAH, since it remains a poorly understood and requires further investigation.

**Methods:**

A single-center cohort study was carried out with all hospitalizations for spontaneous SAH. Patients were classified according to the pattern of bleeding at cranial computed tomography (CT), as those with a PSAH pattern and those with an aneurysmal pattern of bleeding (NPSAH). A DSA was performed in all patients, and only those who showed no vascular abnormalities were included for data collection. Clinical, epidemiological and functional data were collected and analyzed, comparing the results between both groups.

**Results:**

A total of 550 patients with spontaneous SAH were treated at our center, and 74 were classified as nonaneurysmal and included in the final analysis. The bleeding pattern was classified as PSAH in 20 patients and as NPSAH in 54. The NPSAH was more prevalent in women, and PSAH in men. Rates of hydrocephalus and vasospasm were higher in patients with the NPSAH pattern, and clinical outcomes were worse.

**Conclusion:**

The NPSAH pattern had an intermediate clinical behavior between perimesencephalic and aneurysmal bleeding. On the other hand, PSAH had a benign course and excellent prognosis.

## INTRODUCTION

The majority of spontaneous subarachnoid hemorrhages (SAH) are caused by aneurysm rupture. 1 2 Its management is based on aneurysm treatment and its complications. However, around 15% of spontaneous SAH cannot be attributed to aneurysm rupture and are classified as non-aneurysmal , which can be perimesencephalic (PSAH) or non-perimesencephalic (NPSAH). The importance of this subgroup of spontaneous SAH is that the clinical behavior, management, complications and outcomes are different from the aneurysmal type. 3 4 5 6 7 8 Even the different bleeding patterns of non-aneurysmal SAH can vary in clinical behavior and related complications, whether due to a more aggressive clinical course or the failure to identify vascular abnormalities on imaging studies, such as very small aneurysms or arteriovenous malformations. 9

Considering SAH as the smaller group of cerebrovascular diseases and the non-aneurysmal subtype as an even minor subgroup of patients, it is at risk of a non-individualized management. The reference centers of SAH management, based on their great volume of cases, can provide data on the course and outcomes of this particular subtype. 10

In this study we present the complications and outcomes of non-aneurysmal SAH cases, cared for with the same protocols in the same hospital, during around 7 years, comparing the cases of PSAH and NPSAH.

## METHODS

A single-center cohort study was carried out from September 2016 to March 2024, with all hospitalizations for spontaneous SAH in the Cristo Redentor Hospital, Porto Alegre, Brazil. This is a tertiary referral center for neurocritical care, managing approximately 80 cases per year of SAH. All patients in this study were treated using standardized protocols for SAH over a 7-year period.

Data collection was performed through the hospital's electronic medical record system. Initially, all hospitalizations for SAH diagnosed by cranial computed tomography (CT) were included. Patients who did not undergo digital subtraction angiography (DSA) were excluded from the analysis because they did not have vascular investigation with what is considered the gold standard. Thus, only patients with SAH who had vascular investigation with DSA were included for the analysis. These patients were divided according to the findings of the DSA into aneurysmal and non-aneurysmal bleeding.

**Figure 1:**
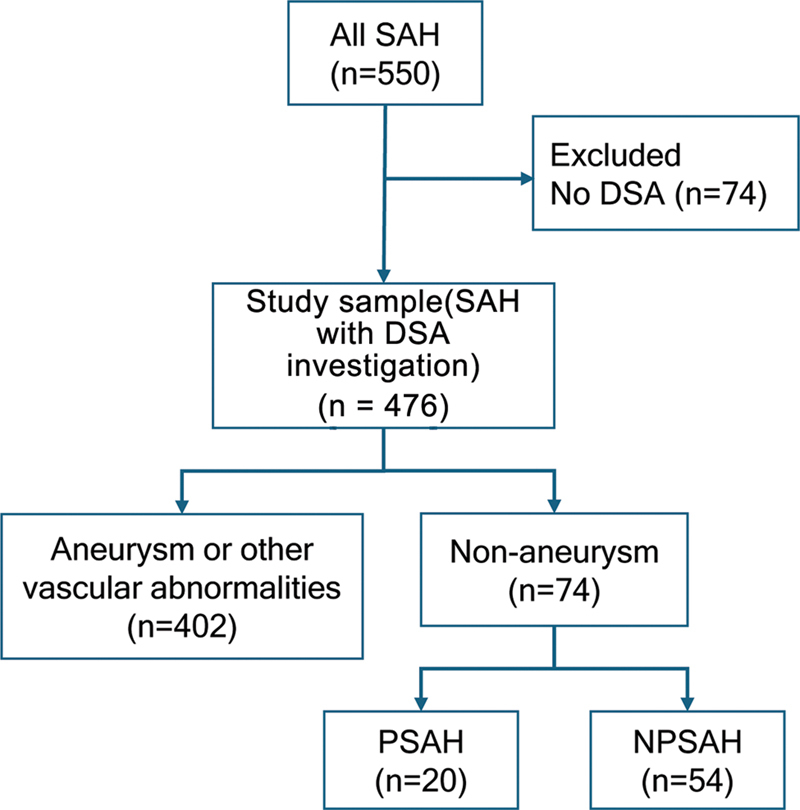
Flowchart of patients included in the present study.

Afterwards, patients with non-aneurysmal bleeding were classified according to the pattern of bleeding at cranial CT, between those with perimesencephalic and aneurysmal patterns, thus becoming the population from which the data were collected. Patients with other vascular malformations were also not analyzed. ( Figure 1 ).

### Definition of perimesencephalic subarachnoid hemorrhage and non-aneurysmatic, non-perimesencephalic, subarachnoid hemorrhage

The perimesencephalic pattern of bleeding was defined according to the Rinkel et al. 11 criteria: SAH epicenter is anterior to the midbrain or pons with possible extension to the perimesencephalic cisterns. It is acceptable for the SAH to extend to the anterior interhemispheric fissure, to the basal part of the Sylvian fissure or small amounts of blood in the occipital horn of the lateral and fourth ventricle.

The aneurysmal pattern of bleeding was defined as the presence of blood in sites often affected by aneurysmal SAH, including the basal cisterns, interhemispheric fissure, Sylvian fissure, intracerebral hemorrhage, and intraventricular space beyond the limits set for the perimesencephalic pattern.

### Digital subtraction angiography (DSA)

Only patients with SAH who underwent DSA, after the diagnosis of SAH by cranial CT, were included for analysis. Subsequently, those with two negative DSA exams were then considered non-aneurysmal SAH. This was the examination of choice for patient selection because it is considered the gold standard for investigating intracranial vascular lesions. Patients with non-aneurysmal SAH were then divided into PSAH and non-aneurysmal, non-perimesencephalic SAH types, according to the CT pattern.

All DSA examinations included bilateral internal and external carotid arteries, and vertebral arteries. If the first DSA is negative for vascular changes, it is repeated in 2 weeks, usually after the period of vasospasm, which could mask smaller aneurysms. The time to repeat the DSA may vary a few days depending on the patient's clinical condition or other logistical factors.

### Data collection

We recorded the following variables: gender, age, ethnicity, comorbidities (diabetes, hypertension, and smoking), World Federation Neurological Surgery (WFNS) scale, Hunt & Hess scale (HH), modified Fisher scale (mFS), presence of vasospasm on DSA, transcranial Doppler (TCD) or angiotomography, placement of an invasive intracranial pressure (ICP) monitoring or external ventricular drain, presence of cerebral ischemia, seizure, or hyponatremia. Functional outcomes were assessed during hospital discharge, according to the modified Rankin scale (mRS). A good outcome was considered as a mRS of 0 to 2, while an mRS > 2 was considered poor. After data collection, the results between PSAH and NPSAH were compared.

The primary outcome of the study was the presence of vasospasm, cerebral ischemia, need for external ventricular drain, or intracranial pressure monitoring, and the functional outcome, assessed through the mRS. Secondary outcomes included the presence of seizures and hyponatremia.

### Statistical analysis

The descriptive analysis of categorical variables was performed using absolute and relative frequencies. Continuous variables with a normal distribution were summarized using the mean and standard deviation (SD), whereas those without a normal distribution were described using the median and interquartile range (IQR), with 25th and 75th percentiles. The Shapiro–Wilk test was used to assess the normality of continuous variables.

Comparisons of categorical variables between the PSAH and NPSAH groups were conducted using Pearson's chi-squared test or Fisher's exact test, as appropriate. Comparisons of scale variables were performed using the Mann–Whitney U test. Statistical analyses were carried out using IBM SPSS Statistics for Windows (IBM Corp.), version 20.0. The significance level adopted was 0.05.

### Approvals

Clarifications and guidance regarding the purpose of the research, as well as authorization from the patient or family member to participate, were provided via telephone. This project has been approved by the institutional review board, under the CAAE number 27362719.6.0000.5530, approval opinion number 6.257.784.

## RESULTS

A total of 550 patients with SAH were included in the database, 476 of whom underwent DSA and were included for analysis, with 16% (n = 74) having no evidence of vascular malformation. The mean time between DSA to classify it as a non-aneurysmal SAH was 13 days. The bleeding pattern was classified as perimesencephalic in 20 patients and as non-perimesencephalic in 54 ( Figure 1 ). Data collection is presented in Table 1 and some of the results have been summarized in Table 2 .

**Table 1:** Demographic and clinical data

SAH pattern	PSAH (n = 20)	NPSAH (n = 54)	*p* -value
Demographic characteristics*			
Male sex,* n (%)	15 (75%)	22 (40.7%)	0.01
Mean age, years	53	54	
Ethnic group* White Black Other	19 (95%) 1 (5%) 0	43 (79.6%) 8 (14.8%) 3 (5.6%)	0.262
Comorbidities*			
Diabetes, n (%)	1 (5%)	5 (9.3%)	0.551
Hypertension, n (%)	8 (40%)	29 (53.7%)	0.295
Smoking, n (%)	6 (30%)	19 (35.2%)	0.675
**WFNS distribution**			0.01
1	18 (90%)	20 (37%)	
2	1 (5%)	8 (14.8%)	
3	0	5 (9.2%)	
4	0	6 (11.1%)	
5	1 (5%)	15 (27.7%)	
**HH distribution**			0.02
1	5 (25%)	10 (18.5%)	
2	13 (65%)	16 (29.6%)	
3	0	8 (14.8%)	
4	0	9 (16.6%)	
5	2 (10%)	11 (20.3%)	
**mFS distribution**			0.01
0	0	3 (5.5%)	
1	2 (10%)	2 (3.7%)	
2	5 (25%)	5 (9.2%)	
3	11 (55%)	18 (33.3%)	
4	2 (10%)	26 (48.1%)	
ICP monitoring*	2 (10%)	7 (12.9%)	0.729

Abbreviations: HH, Hunt-Hess; ICP, intracranial pressure; mFS, modified Fisher scale; NPSAH, non-perimesencephalic SAH; PSAH, perimesencephalic SAH; SAH, subarachnoid hemorrhage; TCD, transcranial Doppler.

**Table 2:** Complications of different patterns of SAH

SAH pattern	PSAH	NPSAH	*p-* value
Number of patients	20 (27%)	54 (73%)	–
Hydrocephalus	2 (10%)	13 (24%)	0.328
Vasospasm at TCD	0	12 (22%)	0.029
Cerebral ischemia	0	4 (7.4%)	0.569
Hyponatremia	1 (5%)	10 (18.5%)	0.269
Seizure	1 (5%)	7 (13%)	0.435

Abbreviations: NPSAH, nonperimesencephalic SAH; PSAH, perimesencephalic SAH; SAH, subarachnoid hemorrhage; TCD, transcranial Doppler.

### Patient characteristics

#### *Gender, age, ethnicity, and comorbidities*

Most of the patients with PSAH were men (75%), while those with NPSAH were women (60%), with a statistically significant difference (I = 0.018). The mean age was similar between the groups, with a mean of 54 years for NPSAH and 53 years for PSAH ( *p* = 0.745). Regarding ethnicity, the vast majority were white, both the patients with PSAH and NPSAH (95 and 80% respectively, I = 0.454). Diabetes, smoking, and hypertension were more prevalent in patients with NPSAH; however, the difference was small and without statistical significance ( *p* = 1.000, 0.786 and 0.433, respectively).

#### *Clinical and tomographic presentation (WFNS, HH, and mF)*

Both the WFNS scale and HH's results were statistically different between the groups, with a median of WFNS 1 (IQR: 1.0–1.0) in patients with PSAH, and WFNS 2 (IQR: 1.0–5.0) in NPSAH ( *p* < 0.001). The HH scale had a median of 3 (IQR: 2.0–4.0) in NPSAH and 2 in PSAH (IQR: 1.25–2.0; *p* = 0.020). The median of the mFS was 3 in both the PSAH (IQR: 2.0–3.0) and NPSAH (IQR: 3.0–4.0) groups ( *p* = 0.013).

### Primary outcomes

#### *Vasospasm and cerebral ischemia*

On the initial DSA, patients with PSAH did not show signs of vasospasm, while 14% of the NPSAH group did ( *p* = 0.167). When using TCD and CT angiography, the difference between the groups was statistically significant. Again, no patients with PSAH showed signs of vasospasm in these exams, while 22% of patients with NPSAH did ( *p* = 0.029). A minority of patients presented cerebral ischemia on CT, with only 4 cases (7.5%) of patients with NPSAH and none with PSAH ( *p* = 0.569).

#### *External ventricular drain and intracranial pressure monitoring*

The use of an external ventricular drain to manage hydrocephalus was necessary in 24% of patients with NPSAH, and in 10% of patients with PSAH ( *p* = 0.328). Intracranial pressure monitoring was performed in 13% of patients with NPSAH and in 10% of patients with PSAH ( *p* = 1.000). Of the patients who required ICP monitoring, two had increased ICP at some point, both in the NPSAH group.

#### *Functional outcome at hospital discharge (mRS)*

**Figure 2:**
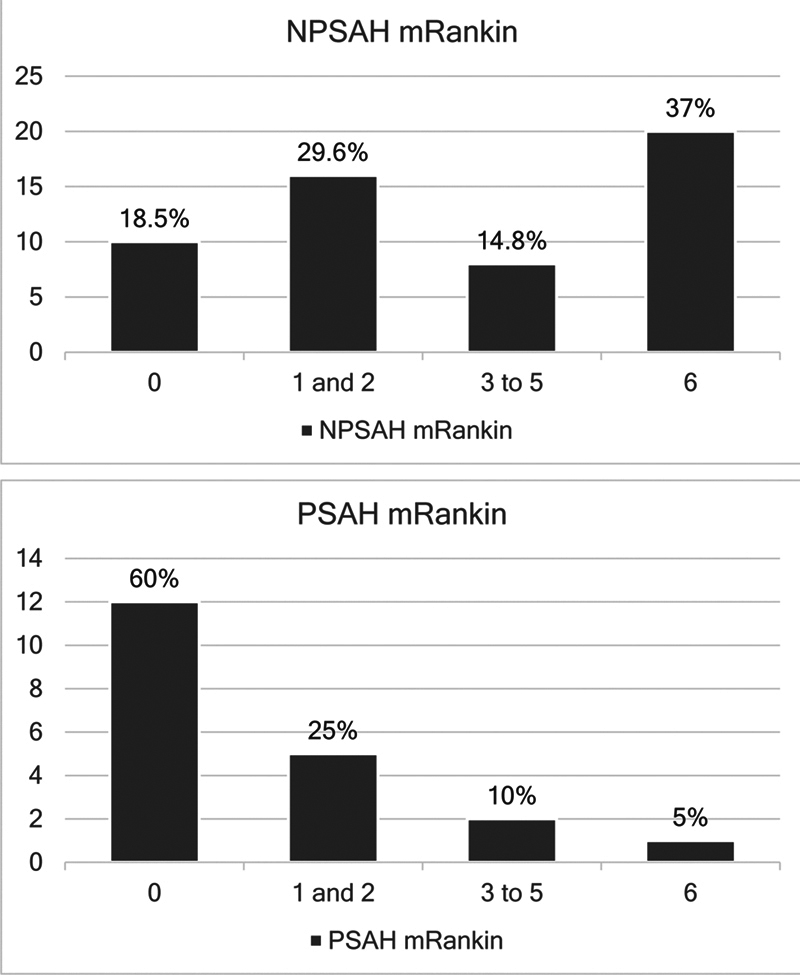
Modified Rankin scale at hospital discharge according to bleeding pattern.

The functional outcome differed greatly between patients with NPSAH and PSAH. Patients with PSAH had a median of 0 (IQR: 0.0–1.0) on the mRS scale at hospital discharge, while the NPSAH group had a median of 4 (IQR: 1.0–6.0; *p* < 0.001). In the PSAH group, 85% of patients had a good functional outcome (mRS 0 to 2), compared to only 48% of the NPSAH group. The distribution of patients on the modified Rankin scale was represented in Figure 2 .

### Secondary outcome

#### *Clinical complications (seizure and hyponatremia)*

The seizure rate was low in both groups, being slightly higher in patients with NPSAH, with 7 patients having had a seizure, while only 1 in the PSAH group ( *p* = 0.435). Similarly, only 10 patients in the NPSAH group presented hyponatremia, and 1 in the PSAH group ( *p* = 0.269).

## DISCUSSION

Up to 15% of cases of spontaneous SAH are non-aneurysmal (no vascular malformations on DSA studies), being typically considered a disease with a better prognosis. Nonaneurysmal SAH can follow a typical bleeding pattern called PSAH, a well-described and studied form of intracranial bleeding. On the other hand, it can have a diffuse pattern that does not meet the criteria for PSAH, but also without evidence of vascular malformations, named NPSAH.

The SAHs of nonaneurysmal etiology have a more favorable prognosis when compared to aneurysmal ones, with more than 80% of patients having a favorable outcome. 12 13 14 In our study, we evaluated the different clinical outcomes of patients with non-aneurysmal SAH according to the tomographic pattern of the hemorrhage, showing that patients with the PSAH pattern have a more favorable course and fewer complications when compared to NPSAH, despite both being of non-aneurysmal origin.

Approximately 7% of SAHs are PSAH, and its incidence has increased over the years, which can be attributed to better description and understanding of the disease, increasing the diagnosis rates. 15 Criteria for diagnosing a PSAH include epicenter of the SAH anterior to the midbrain or pons, with possible extension to the perimesencephalic cisterns. It is acceptable for the SAH to extend to the anterior interhemispheric fissure, to the basal part of the Sylvian fissure, or to small amounts of blood in the occipital horn of the lateral ventricle and fourth ventricle. 11 Its etiology is not completely understood and may be caused by rupture of deep veins around the midbrain. 16 In the study by Mensing et al., the majority of patients were men (58%), with a mean age of 53 years. 17 Headache is the most common initial symptom, followed by nausea, vomiting, and neck stiffness. Its course tends to be predominantly benign and has an excellent long-term neurological and functional prognosis. 8 18 19 20

The NPSAH is much less studied than PSAH. Its etiology is not well defined, and the diagnostic criteria are simply based on a SAH without evidence of vascular malformation, which does not follow the diagnostic criteria for perimesencephalic presentation. Its clinical pattern appears to be more aggressive than PSAH, despite still being a more benign entity than aneurysmal SAH. 21 22

The incidence of complications and poor outcomes is lower in PSAH when compared to aneurysmal SAH and NPSAH. 22 In aneurysmal bleeding, up to 30% of patients present with hydrocephalus, many acutely requiring ventriculostomy and later ventriculoperitoneal shunt. 23 In PSAH, the incidence of hydrocephalus is approximately 15%, and it rarely requires permanent ventricular shunting. Of the patients who develop hydrocephalus, only 3% will need external ventricular drainage due to reduced level of consciousness. In our study, the ventriculostomy rates for PSAH were 10%. Patients with NPSAH are at increased risk of developing hydrocephalus, with rates up to 30%. 13 17 21

Cerebral vasospasm is one of the most common complications of aneurysmal bleeding, developing in approximately 50% of patients. 24 Vasospasm rates in PSAH have a variable incidence between studies, ranging from approximately 8 to 12%, with the vast majority being asymptomatic and diagnosed by imaging tests. Delayed cerebral ischemia is also rare, with an incidence of 0.7%. 19 Meanwhile, patients with NPSAH had rates of more than 30% of vasospasm and 6% of delayed cerebral ischemia. 25 In our study, no patient with PSAH showed DSA or ultrasonographic signs of vasospasm, while patients with NPSAH had a 20% rate, and only 4 patients had cerebral ischemia on imaging studies. In a sample of patients with aneurysmal bleeding from the same center, the rates of delayed cerebral ischemia were 23%. 6

Clinical and demographic characteristics of PSAH and NPSAH are similar in literature. The mean age reported in the literature is approximately 50 years for both PSAH and NPSAH, with a slightly higher prevalence in females. Headaches are the most common symptom in both types. However, patients with NPSAH more commonly presented changes in consciousness level. 21 25 26 Our study presented ages similar to those reported in the literature, with a mean of 54 years for NPSAH and 53 years for PSAH. There was a higher prevalence of PSAH cases in males. However, cases of NPSAH were more common in females.

The HH and WFNS, which are clinical assessment scales, had low (grade 1 or 2) results in the vast majority of patients in both groups, according to Coelho et al., although patients with NPSAH (HH 1-2: 75%, WFNS 1-2: 78%) had lower rates compared to PSAH (HH 1-2: 89%; WFNS 1-2: 93%). 25 Higher grades on the clinical HH and WFNS scales for patients with NPSAH were also reported by Bashir et al. 21 In our study, 90% of patients with PSAH had an HH grade of 1 or 2, and 95% had a WFNS grade of 1 or 2. Among patients with NPSAH, only 47% had an HH grade of 1 or 2, and 51% had a WFNS grade of 1 or 2.

Regarding functional outcomes, Coelho et al. showed that 93% of patients with PSAH had an mRS 0 or 1 after more than 1 year of follow-up, compared to 85% of patients with NPSAH, which could mean a more aggressive clinical course in these cases. 25 Similar findings were described in a meta-analysis by Boswell et al. 22 Our series demonstrated higher rates of unfavorable outcome for patients with NPSAH, with a mean of 4 on the mRS. Patients with PSAH also had a mean mRS of 0. A good functional outcome (mRS 0 to 2) was seen in 85% of patients with PSAH, and in 48% with NPSAH.

The higher rates of poor outcomes in the NPSAH group may indicate a more aggressive clinical behavior of this pattern of hemorrhage, due to factors that are not yet explained, which may be related to the distribution of bleeding in the subarachnoid space, a different pathophysiology than PSAH, or even vascular changes not identified in imaging exams, such as an aneurysmal blister, small arteriovenous fistulas, undetected arteriovenous malformations and even spinal vascular malformations, which may go unnoticed in a cerebral SAH. The presence of an undetected vascular abnormality may explain the intermediate behavior of NPSAH when compared to aneurysmal SAH and PSAH, since the bleeding caused by a vascular alteration would have a pathophysiology more similar to the aneurysmal bleeding. In this context, the search for vascular abnormalities should be carried out more cautiously, using different diagnostic modalities, repeating examinations more frequently and improving the DSA technique. 27

A retrospective study published by Tarkiainen et al. 26 presented a cohort study of 214 patients with non-aneurysmal SAH. When comparing PSAH with NPSAH, they found that the vast majority of patients had a favorable clinical outcome. However, of the patients who had an unfavorable outcome, the majority belonged to the NPSAH group, denoting an intermediate clinical course for this type of pathology. In their study, 22% of patients with NPSAH required ventriculostomy, a slightly lower percentage than that found in our study, which was 24%. As in our study, ventriculostomy rates in these patients were higher than those in PSAH. Also, mFS grade 3 was the most common bleeding pattern, as well as vasospasm rates, which were present in 20% of cases, similar to those reported in previous studies. 12 28 29

### Limitations

The present study has some limitations. The low sample size made it impossible for some of the variables to reach statistical significance. The functional outcome was only assessed at hospital discharge, since there was loss of follow-up of patients for a long-term analysis, which could show an improvement in the mRS in patients with NPSAH.

In conclusion, in the present study, patients with an NPSAH experienced more complications resulting from hemorrhage with a significant impact on prognosis compared to PSAH. The need for ventriculostomy and vasospasm was higher for cases of NPSAH than for PSAH, and lower when compared to bleeding of aneurysmal origin data, showing an intermediate clinical behavior between perimesencephalic and aneurysmal bleeding. On the other hand, PSAH had a benign course and excellent prognosis. One possible explanation for worse outcomes in patients with NPSAH could be the presence of unidentified vascular abnormalities, such as blister aneurysms, small arteriovenous fistulas, or small arteriovenous malformations.
